# Supplementary material for: Critical Parameters in Dynamic Network Modeling of Sepsis
Source: Front Netw Physiol. 2022 Jun 1;2:904480. doi: 10.3389/fnetp.2022.904480 (PMC10012967; doi:10.3389/fnetp.2022.904480)
Supplement: Supplementary file 1 [file DataSheet1.PDF]

Supplemental Material:
Critical parameters in dynamic network modeling of sepsis

Rico Berner[1,2], Jakub Sawicki[2,3,4], Max Thiele[2], Thomas Löser[5], and Eckehard Schöll[2,3,6]

[1]Institut für Physik, Humboldt-Universität zu Berlin, Newtonstraße 15, 12489 Berlin, Germany
[2]Institut für Theoretische Physik, Technische Universität Berlin, Hardenbergstraße 36, 10623 Berlin, Germany
[3]Potsdam Institute for Climate Impact Research, Telegrafenberg A 31, 14473 Potsdam, Germany
[4]Fachhochschule Nordwestschweiz FHNW, Leonhardsstrasse 6, 4009 Basel, Switzerland
[5]Institut LOESER, Wettiner Straße 6, 04105 Leipzig, Germany
[6]Bernstein Center for Computational Neuroscience Berlin, Humboldt-Universität, Philippstraße 13, 10115 Berlin, Germany

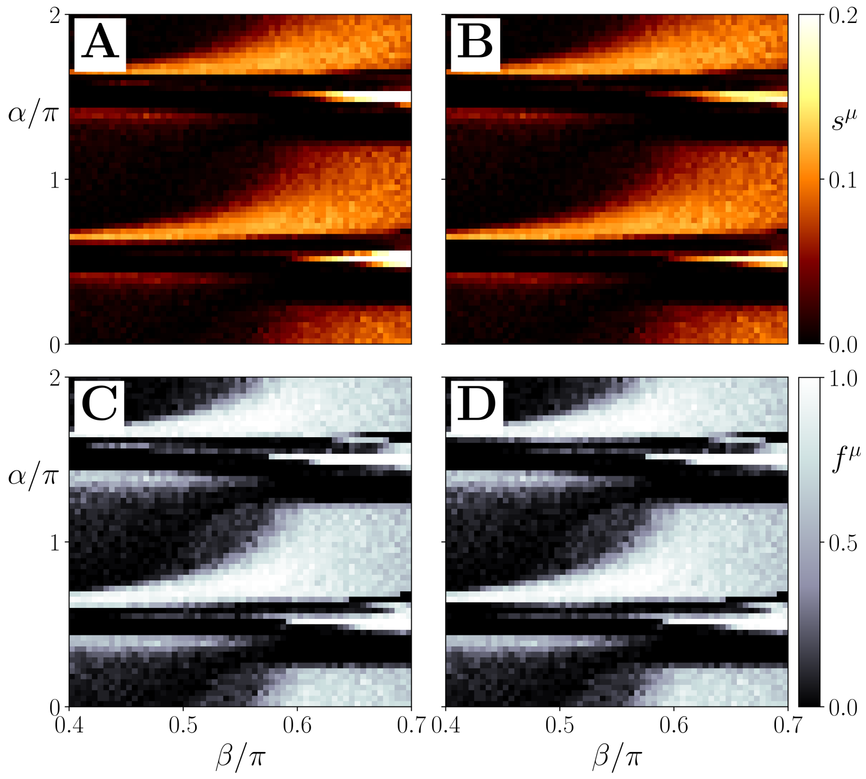

Figure S.1: Map of regimes: ensemble average s^μ of the standard deviation of the spatially averaged mean phase velocities (top panels) and the frequency cluster ratio (bottom panels) in the parameter plane of age parameter β and interlayer interaction phase lag α for the parenchymal (left column) and immune layer (right column), respectively. Bright colors correspond to the formation of frequency clusters. Ensemble size is $N_E = 50$. Simulation parameters: $\sigma = 1$, $\alpha \equiv \alpha^{12} = \alpha^{21}$; all other parameters as in Figure 3.
